# Patients' Experiences of Multimodal Rehabilitation for Chronic Pain in Primary Care

**DOI:** 10.1002/msc.70241

**Published:** 2026-06-25

**Authors:** Charlotte Sylwander, Josefin Willhammar, Ingrid Larsson, Stefan Bergman

**Affiliations:** ^1^ School of Health and Welfare Halmstad University Halmstad Sweden; ^2^ Spenshult Research and Development Centre Halmstad Sweden; ^3^ Department of Clinical Sciences Section of Rheumatology Lund University Lund Sweden; ^4^ Family Medicine School of Public Health and Community Medicine Institute of Medicine Sahlgrenska Academy University of Gothenburg Gothenburg Sweden

**Keywords:** multidisciplinary treatment, multimodal rehabilitation, musculoskeletal pain, primary care, qualitative study

## Abstract

**Objectives:**

To describe how patients with chronic pain experienced participation in a multimodal rehabilitation programme (MMR) in primary care.

**Methods:**

The study had a descriptive qualitative design with an inductive approach, including 10 participants (6 women and 4 men, aged 36–57). The MMR took place at a primary care centre in southern Sweden and consisted of face‐to‐face meetings twice a week for 6 to 7 weeks. The multidisciplinary team included a physiotherapist, an occupational therapist, a healthcare counsellor, and a physician. Individual in‐person interviews were conducted using open‐ended questions and analysed using qualitative content analysis.

**Results:**

The results illustrate participants' varied experiences of participation in MMR in primary care, described in three themes with six sub‐themes: (1) knowing the pain (grasping the pain and voicing needs); (2) embracing pain (being recognised and empowered); and (3) building resilience (enhancing skillset and becoming proactive).

**Conclusions:**

MMR offered a structure to address chronic pain beyond symptoms, fostering better communication with healthcare professionals and promoting autonomy. It encouraged a shift towards a biopsychosocial understanding of pain and resilience building. Tailoring MMR to individual needs and previous experiences could further strengthen its impact on diverse groups of patients with chronic pain.

## 1 Introduction

Chronic musculoskeletal pain, commonly defined as pain persisting for 3 months or longer (Treede et al. 2019), is highly prevalent and affects nearly one‐third of the global adult population (Cohen et al. 2021). It is also a leading reason for seeking healthcare (Cohen et al. 2021), and musculoskeletal problems account for approximately 20% of visits to Swedish primary healthcare facilities (The National Board of Health and Welfare 2021).

Chronic pain affects various aspects of an individual's life, including physical, mental, and social well‐being, as explained by the biopsychosocial pain model (Holopainen 2021). It is associated with poorer overall health outcomes (Holopainen 2021) and increased sickness absence rates (Harker et al. 2012; Schneider et al. 2010). Chronic pain is also associated with increased levels of anxiety, feelings of helplessness, loneliness, independence, and depression (Järemo et al. 2017; MacNeela et al. 2015; Mills et al. 2019; Westergården et al. 2021), which in turn are linked to a poorer recovery prognosis (Mills et al. 2019). Individuals with chronic pain describe perceiving their bodies as obstacles, which contributes to substantial struggles and limitations in performing everyday tasks and leisure activities (MacNeela et al. 2015; Sylwander et al. 2022; Toye et al. 2017; Westergården et al. 2021). Moreover, the pain can persist for decades (Sylwander et al. 2020), resulting in prolonged impairment of health, daily functioning, and occupational engagement (Sylwander et al. 2020; Toye et al. 2017; Wainwright et al. 2022). Chronic pain can also lead to an experienced loss of independence and previous social roles, social isolation, disempowerment, and diminished personal self‐worth (MacNeela et al. 2015).

A multimodal pain rehabilitation programme (MMR) is based on the biopsychosocial model (Engel 1981; Holopainen 2021) and aims to address the multifaceted nature of chronic pain across biological, psychological, and social domains. A multidisciplinary team delivers MMR of healthcare professionals (HPs) who collaborate to provide combined interventions tailored to the individual needs of each patient (Gatchel et al. 2014). Separating MMR between primary and specialist care enhances resource efficiency: individuals with less complex pain conditions can receive MMR in primary care, while MMR in specialist care is intended for those with particularly complex chronic pain conditions, often accompanied by more pronounced psychological comorbidity (National Medical Indications 2011).

Previous research has found MMR in primary care to be a cost‐effective (Eklund et al. 2021) and efficient treatment compared to usual unimodal care, in terms of pain relief, improved functional capacity, and returning to work (Kamper et al. 2015; Kechichian et al. 2022). Similar results have been found in Sweden: 1 year after conducting MMR, positive effects were observed on physical and emotional function, health‐related quality of life, and sick leave was reduced (Falkhamn et al. 2023; Pietilä‐Holmner et al. 2020). However, attendance varies, and patient experiences underpinning programme completion, differential treatment outcomes, and observed inequalities in benefit remain poorly understood (Falkhamn et al. 2023; Gerdle et al. 2020; Pietilä‐Holmner et al. 2020). There is thus a need to further develop the MMR delivered in primary and secondary care. Therefore, this study aimed to describe how patients with chronic pain experienced participation in a multimodal pain rehabilitation programme in primary care.

## 2 Methods

### 2.1 Study Design

The study had a descriptive qualitative design with an inductive approach to describe individuals' experiences of participation in an MMR programme (Graneheim and Lundman 2004). The Consolidated Criteria for Reporting Qualitative Research (COREQ) 32‐item checklist was followed to ensure trustworthiness (Tong et al. 2007).

### 2.2 Setting

The MMR was delivered at primary care clinic in southern Sweden and patients were included according to the national criteria: aged 18–65; with disabling chronic pain having lasted for more 3 months (neck, shoulder, back or widespread), currently on sick leave or with significant disruption to daily activities due to the pain, with no other diseases or conditions that could conflict with the MMR (National Medical Indications 2011). The individuals were to have undergone pharmacological and non‐pharmacological unimodal rehabilitation (e.g., physiotherapy) without achieving an acceptable effect. The MMR was available to all patients who fulfilled the criteria, independent of socioeconomic status.

The multidisciplinary team consisted of a physiotherapist, an occupational therapist, a healthcare counsellor, and a general practitioner, all with competence in complex chronic pain. The overarching goal of the MMR is to equip participants with strategies to cope with the impact of chronic pain while strengthening their capacity to function across various social settings (National Medical Indications 2011).

The programme consisted of group‐based sessions twice a week over six to 7 weeks. Each session lasted approximately 2 h. The MMR provided in primary care offers person‐centred goal setting and cognitive behavioural therapy (CBT) for pain management, including pain education (basic pain physiology, stress and emotional aspects), and CBT for sleep problems (National Medical Indications 2011). The programme further includes physical activity and functional training with professional coaching (including activities of daily living) and optimising environmental factors by identifying physical and social factors that negatively affect the individual's pain. Lastly, it includes sustaining management strategies and relapse prevention. The MMR provided in specialist care also includes CBT for anxiety and depression, graded exposure in vivo, and tailored interventions for comorbidity (National Medical Indications 2011).

### 2.3 Participants

Individuals with chronic pain who completed the MMR programme in spring 2017 or autumn 2018 were invited to participate within 1 month of completion (*n* = 14). Ten individuals were accepted, five from each occasion, and included in the study (six women and four men, aged 36–56). Four participants had a first language other than Swedish but were able to participate without an interpreter. The four individuals who declined to participate provided no reason, and no further questions were asked. Sample adequacy was considered in terms of information power, which suggests that the more relevant information a sample provides to the study aim, the fewer participants are needed (Malterud et al. 2016). The study had a focused aim, and all participants had recent and direct experience of the same MMR programme.

### 2.4 Data Collection

Individual, in‐person interviews were conducted by a medical doctor (J.W.JW (medical doctor) in a private room at the primary care clinic. The interviewer was part of an MMR team but not the one assigned to study participants, had no prior relationship with the participants, and adopted a participatory and reflective approach, asking follow‐up questions when responses required further elaboration (Brinkmann and Kvale 2015). The interviews were guided by open‐ended questions exploring experiences of the MMR and the team, such as ‘How have you experienced the MMR programme?; ‘How did you experience the contact with the team?; and ‘How do you experience your ability to change your life situation after the MMR?’. Follow‐up questions were used to deepen the responses, such as ‘Please, can you tell me more about…’ or ‘How do you mean…?’. J.W. had limited prior experience conducting research interviews but extensive experience in taking clinical anamneses. To ensure the quality and relevance of both the interview guide and the interview process, a pilot interview was conducted with the MMR team before data collection, confirming that no adjustments were necessary. The interviews were audio‐recorded and transcribed verbatim, ranging from 16 to 29 minutes (mean 23 minutes).

### 2.5 Data Analysis

Qualitative content analysis was used to examine the transcribed interviews (Graneheim et al. 2017; Graneheim and Lundman 2004). The first author (C.S., senior lecturer, PhD) initially read the interviews to gain an overall understanding and performed the coding. Meaning units relevant to the aim of the study were identified (*n* = 184) and condensed into descriptive codes, maintaining low abstraction and interpretation (Graneheim et al. 2017; Graneheim and Lundman 2004; Lindgren et al. 2020). Data sufficiency was assessed in terms of information power and the relevance, variation, and richness of the material in addressing the study aim, rather than through a formal assessment of saturation (Malterud et al. 2016). Codes were then organised into sub‐themes based on similarities and differences and further grouped into themes (Graneheim et al. 2017). The sorting procedure was conducted collaboratively (C.S., J.W., S.B., professor, MD, PhD, and I.L., professor, RN, PhD) with continuous discussions among all authors (Graneheim and Lundman 2004; Lindgren et al. 2020). Any disagreements were discussed until a consensus was reached. The process was iterative, involving cycles of de‐contextualization and re‐contextualization to ensure analytical depth and rigour (Lindgren et al. 2020). A first draft was presented in a conference abstract (Sylwander et al. 2025), and the analysis continued back and forth until a consensus was reached. The final analysis resulted in six sub‐themes within three overarching themes (Lindgren et al. 2020). C.S., S.B. and I.L. all have previous experience in qualitative analysis. See example from the coding tree in Table 1.

**TABLE 1:** Examples from the coding tree.

Condensed meaning unit	Code	Sub‐theme	Theme
I'm thrilled to have participated in MMR, even though the cause might be a bit sad. But I felt important. (Participant no. 2)	Felt important	Being recognised	Embracing pain
The contact with the team wasn't particularly good. It felt like going back to square one, and it didn't do me any good. (Participant no. 8)	Back to square one	Being recognised	Embracing pain

### 2.6 Ethics

The principles followed in this research are those outlined in the Helsinki Declaration (World Medical Association 2013) and the four ethical principles: beneficence, non‐maleficence, justice, and autonomy (Garrett et al. 2001). Before participation, all individuals received information about the study's aim, procedure, and their rights, including the right to withdraw at any time. Written informed consent was obtained, and the study was approved by the Regional Ethical Review Board in Lund, Sweden (Dnr: 2017/333).

## 3 Results

**FIGURE 1:**
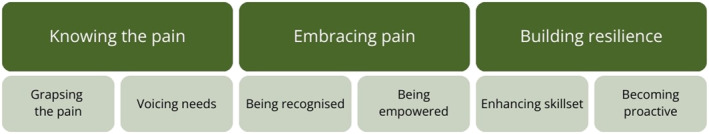
Overview of the results of the patients' experiences of a multimodal rehabilitation program for chronic pain. Presented with three themes and six sub‐themes.

The results describe how patients with chronic pain experienced participation in MMR, expressed as a process of knowing the pain, embracing pain, and building resilience (see Figure 1).

### 3.1 Knowing the Pain

Patients with chronic pain experienced participation in MMR as a process of knowing their pain, which involved both grasping the pain and voicing their needs.

#### 3.1.1 Grasping the Pain

Patients experienced that the MMR provided a deeper understanding of the complexity of chronic pain and the importance of addressing its psychological aspects. For some, this understanding alleviated the pain. They learned to better manage their mental health, tried not to always perform at maximum capacity, and to find a balance in daily life. For others, the pain remained, but grasping its complexity facilitated a process of knowing and relating to the pain. Patients experienced the multidisciplinary team as knowledgeable, with each HP contributing valuable information. The combination of a physiotherapist, occupational therapist, and healthcare counsellor was particularly helpful in grasping the complexity of chronic pain. Activities where the participants were actively engaged and articulated their own experiences were effective in the process of knowing their pain.

They sometimes asked us what we experienced as pain, what we needed in order to feel good, and so on. We had to express our experiences in our own words and then find ways to make it easier.

(Participant no. 3)

Experiences of pain education varied, and not all participants gained new knowledge. Some participants described repetition as helpful, whereas others found the content already known, repetitive, too factual, or insufficiently adapted to their expectations and prior knowledge. A positive outlook was expressed by those who found repetition and reminders useful, as they led to new insights. Others were disappointed, having expected more from the MMR. Group discussions or other participatory elements were preferred over lecture‐based sessions, as these tended to be very factual.

We always had a lecturer or similar, and I already knew that it [the pain experience] looks different [from one person to another]. But I believe that I always took something with me when I left. Something that I had to think about a little. Change something small about myself or do it differently.

(Participant no. 5)

#### 3.1.2 Voicing Needs

Patients with chronic pain found that the MMR enabled them to voice their needs to family, at work, and in their environment. During the MMR, participants shared experiences with others in the group and with family at home and observed a positive change in their ability to talk about pain. Being able to express needs, such as factors essential to managing pain, deepened their knowing of their pain and led to increased understanding, acceptance, and support from others.

My children didn’t accept it [the pain] before. But since I’ve told them about what I experienced here, in the rehabilitation, they understand better. Now it’s my 11‐year‐old who says, Come on! We’re going to find Pokémon; it’s for you. Then we go out for a walk.

(Participant no. 1)

Although some patients with chronic pain experienced that the MMR supported the development of voicing their needs, they wanted more. The sense of not being understood or heard, given the invisible nature of chronic pain, was frustrating, and patients suggested that MMR should include guidance on how to handle such situations. Some expressed frustration at not having received information about MMR earlier, noting that being able to voice needs for appropriate support requires awareness of MMR and other available treatments or services. Moreover, representatives from the Swedish Social Insurance Agency were suggested for inclusion in the future, given the communication challenges in applying for reduced workload and extended sick leave compensation.

I must know what I am entitled to [regarding treatment options] in order to participate. If I don’t, it isn't easy to be involved. And to be listened to [by HPs]; otherwise, it’s also challenging to be involved. So, it’s important to be listened to and receive information. Of course, I can also seek information myself, but not everything.

(Participant no. 9)

### 3.2 Embracing Pain

Patients with chronic pain experienced participation in MMR as a process of embracing their pain, which involved both being recognised and empowered.

#### 3.2.1 Being Recognised

Patients with chronic pain experienced that participation in MMR fostered a sense of recognition, through engagement with others in similar situations, reducing feelings of isolation and facilitating pain acceptance. Learning that chronic pain manifests in different ways, yet often involves shared experiences, was valuable. The MMR supported a sense of calm in accepting that the pain might persist and that embracing it could be more helpful than continuously searching for answers. The team was knowledgeable, empathic, approachable, and available to answer questions. The feeling that the HPs genuinely wanted to listen and respond contributed to the experience of being valued and recognised. The open and friendly environment, where both highs and lows were shared, helped facilitate embracing the pain.

I was able to open up and tell them how I felt, and then I listened to how others felt. There have been good conversations, rewarding conversations. People have listened to me. I've sat and cried, and laughed sometimes. It was a pretty good constellation.

(Participant no. 10)

Patients with chronic pain experienced that the empathetic and respectful HPs reinforced the feeling of being acknowledged, although some reported feeling overlooked and expressed higher expectations. For them, greater insight from the HPs into living with chronic pain would have further strengthened the feeling of being understood and recognised. The participants also appreciated being invited to lead a session themselves.

The last few times, I was responsible for the relaxation [session]. I found it a huge challenge, and it was gratifying. It is more fun when I can give something, if I can feel that there is a need for it, rather than sitting in front of someone in the team who sits and reads word by word and does not know and cannot feel the pain that we have, while sitting on a chair trying to relax. It is almost impossible.

(Participant no. 8)

#### 3.2.2 Being Empowered

Patients with chronic pain experienced enhanced empowerment and a more positive outlook on life through participation in MMR. The group dynamic played a key role by contributing to each other's progress and engaging in meaningful discussions. Participants' optimism and ability to embrace the pain were enhanced. Through participation in MMR, patients found new strength to move forward in life, beginning to overcome previous feelings of depression, isolation, and hopelessness.

One must accept that sometimes things are truly dreadful, and it's perfectly fine to feel sad, have a good cry, and then return to trying and finding your way forward, again. …You need to shift your focus away from dwelling solely on the difficulties and instead try to find the positive aspects, but that's difficult.

(Participant no. 2)

Participants felt encouraged by the HPs to explore new possibilities, which fostered empowerment. Part of the programme was a visit from members of the Swedish Rheumatic Association, who had firsthand experience of chronic pain. It was considered particularly inspiring and helped shift the focus from pain relief to embracing life despite pain. However, excessive negativity and rumination by other participants, together with the persistent nature of pain, hindered a positive outlook for some. The diversity of pain conditions among the participants posed challenges for group cohesion and shared understanding. Those with prior pain rehabilitation experience felt negative discussions about pain worsened their condition, as the programme offered little that was new and failed to meet expectations. The participant suggested that more frequent sessions with informal talks, such as coffee breaks, could increase trust building and openness, increasing the benefits of MMR. The group format was experienced in contrasting ways. For some participants, sharing experiences supported empowerment, whereas for others, discussions characterised by negativity or rumination made it more difficult to maintain a positive outlook.

Sitting around whining and feeling sorry for myself, and wanting others to feel sorry for me, doesn't help me in this situation I'm in, with my pain. It just makes it worse when people pat me on the arm and say, ‘How are you? How are you feeling today?’ Then you feel even worse, like you have to deal with that too.

(Participant no. 8)

### 3.3 Building Resilience

Patients with chronic pain described their MMR participation as building resilience through enhancing their skillset and becoming more proactive.

#### 3.3.1 Enhancing Skillset

During participation in MMR, the patients with chronic pain developed various skills that helped build resilience in managing pain in everyday life. Initially, small tips seemed insignificant, but participants recognised their value when they applied them over time. The focus was not solely on pain but also on daily living, which was considered a positive factor of the MMR. Participation in MMR was described as an awakening to how the accumulated advice made everyday life with pain more manageable.

It [MMR] hasn't just been about pain. It included everything to do with daily life. Everything from making the bed to walking the dog, shopping, etc. It has included a lot, not just focusing on the pain but on ordinary life as well.

(Participant no. 10)

Patients with chronic pain found learning to prioritise self‐care and balance activities to be valuable. MMR contributed to respecting limits due to the pain and taking breaks. Enhancing their psychological skillset was considered a breakthrough in building resilience and coping with pain. MMR encouraged exploring various techniques to facilitate everyday life and ease pain. Participants realised that focusing on distractions from pain, even for brief moments, such as relaxation and finding joy in the small things, had a significant impact. But not all participants experienced built resilience. Some experienced that the facilitation of skill development provided by the MMR was already familiar to them, given their previous experiences with other pain rehabilitation programmes. For these participants, the activities and pain management tips were experienced as too basic and of limited usefulness.

I have learned that you shouldn’t give 100%. You should do as much as possible, but not more than needed.

(Participant no. 7)

#### 3.3.2 Becoming Proactive

MMR promoted various forms of proactivity, from reducing workload to maintaining a positive outlook. Some participants recognised their role in pain management, taking responsibility for engaging in physical activity and regulating their mindset. MMR fostered an approach to building resilience, understanding that it could take time, while the vision of influencing their future increased self‐confidence and empowered them to act. Some began to challenge themselves by attempting activities they previously believed were unhelpful or harmful. After participation in MMR, participants became more open‐minded to activities and ways of thinking that could ease the pain, partly due to the information shared by HPs and group discussions. Some realised how easy it was to become self‐focused and consumed by the pain, but with a more proactive mindset, pain became more manageable, and life easier. Additionally, sleep improved with increased physical activity based on the advice given, further supporting continued proactivity.

I will try the tips and help I got to see if it's good for me. For example, I have got to try that acupressure mat. I'm not used to it, but I'd like to try it because it has helped someone, so maybe it will help me too.

(Participant no. 4)

However, not everyone experienced that MMR influenced proactivity in building resilience. Some participants perceived no improvement in proactivity beyond what they had already achieved in previous treatments, leaving them at the same stage of pain management as before taking part in MMR. This feeling was associated with the experience of being unable to change their situation, aside from possibly making each day a bit easier. Experiences of becoming proactive also varied. Some participants described new ways of acting and managing everyday life with pain, whereas others experienced little change beyond strategies already developed through previous treatments.

I can't change anything, just make the day a little easier.

(Participant No. 7)

## 4 Discussion

This study describes how patients with chronic pain experienced their perception and management of pain after participating in an MMR programme in primary care. The results revealed that MMR facilitated knowing pain by enabling participants to grasp their pain and voice their needs, embrace the pain through recognition and empowerment, and build resilience by enhancing their skillset and developing proactivity. These findings highlight a spectrum of positive to negative experiences of MMR.

### 4.1 Knowing the Pain

MMR fostered a deeper understanding of the complexity of chronic pain, not only in terms of pain‐related knowledge, but also the ability to *grasp the pain's* complexity based on individual context. Understanding how psychological factors impact pain was essential, though some participants had not recognised this connection before the programme. These results align with previous studies describing the importance of pain knowledge, especially letting the participants explain in their own words and tell their stories (Hållstam et al. 2015; Pietilä Holmner et al. 2018; Watson et al. 2019). Pain education alone is not enough; the information needs to be contextualised for individuals to truly grasp the pain. For example, reducing self‐doubt and recognising that pain is not ‘made up’ but genuinely influenced by psychological factors such as attitudes and beliefs, seems to be a key factor in successful MMR in this and similar studies (Hållstam et al. 2015; Battin et al. 2022). Being able to change individuals' attitudes and beliefs is essential to improving quality of life (Mills et al. 2019).

The multidisciplinary team shared helpful information, but it could be perceived as too repetitive, factual, and lacking flexibility. These results align with previous research identifying experiences such as perception of repetition (Pietilä Holmner et al. 2018). Embracing a more holistic perspective of the biopsychosocial model has been identified as an obstacle among HPs (Ng et al. 2021). Another barrier to grasping new disease‐related knowledge is limited health literacy (Mackey et al. 2016). Therefore, MMR in primary care could benefit from implementing or improving health literacy responsiveness (Cesar et al. 2022).

MMR helped participants feel more confident in *voicing their needs* to family, peers, HPs, etc. This improved communication led to greater understanding, acceptance, and support from others. Other studies have not clearly expressed the improved communication skills as a result of MMR. Instead, findings have been embedded in terms of fear of not being understood by peers or being a burden (Battin et al. 2022), being discredited (Pietilä Holmner et al. 2018), or that support from peers was important (Hållstam et al. 2015). A fear of peers not understanding the importance of such a programme has also been discussed (Battin et al. 2022). This fear reflects the broader difficulty many face in communicating their pain‐related needs, yet voicing these needs is essential for receiving support and maintaining quality of life with chronic pain (Sylwander et al. 2020). In addition, unsettling working conditions can increase the pain and load on the individual (Wainwright et al. 2022); not being able to communicate this properly could cause a negative cycle of pain and impaired health. Since poor communication can perpetuate cycles of pain and impaired health, improving communication skills may enhance autonomy by enabling individuals to advocate for their needs, making it an essential outcome of MMR in primary care.

In the current study, there was frustration about not being told about MMR sooner, highlighting the importance of individuals knowing what support and treatment are available. HPs have expressed difficulties with inclusion (Stenberg et al. 2016), and further training in patient selection for MMR in primary care—as opposed to specialised care—has been suggested (Pietilä‐Holmner et al. 2020). HPs have also expressed the importance of patients having positive beliefs in order to gain positive results from MMR and embrace the pain (Stenberg et al. 2016).

### 4.2 Embracing Pain

Taking part in MMR helped individuals feel understood by connecting with others who also live with chronic pain. Reduced feelings of isolation and *being recognised* made it easier to embrace the pain. Participants appreciated learning how pain affects individuals differently while recognising their shared experiences. The supportive and empathetic HPs were crucial to the feeling of being recognised. These results are in line with previous research reporting experiences of being believed and validated (Hållstam et al. 2015; Pietilä Holmner et al. 2018; Battin et al. 2022; Semedo et al. 2020), which are important given that individuals with chronic pain often experience that HPs question their credibility (Samulowitz et al. 2018). On the other hand, some with past treatment experience felt their needs were missed, which hindered them in embracing the pain. These results have been found in previous studies, especially among those with previous negative experiences of healthcare (Battin et al. 2022).

MMR *empowered* individuals to be more positive and in control. Participants supported one another through honest conversations, building a sense of community and helping each other see things from different angles. Including members of the Swedish Rheumatic Association, who also live with chronic pain, was especially inspiring. Previous research has found similar results, where the feeling of not being alone or realising that others are worse off helps one embrace the pain (Hållstam et al. 2015; Semedo et al. 2020). The experience of optimism and hope is associated with reduced pain and dysfunction (Basten‐Günther et al. 2019; Shanahan et al. 2021). On the contrary, having emotional distress can negatively impact pain [40], which could explain why some participants responded adversely to group discussions characterised by rumination, in their opinion, and as a result, were not empowered or could not embrace the pain. Previous research has identified feelings of repetition and even setbacks among those with previous treatment experience (Pietilä Holmner et al. 2018), suggesting that MMR in primary care might be best suited for individuals who have not received any similar prior treatment. Moreover, negative beliefs could be due to low self‐efficacy (an individual's belief in their capacity to achieve desired outcomes [44]) and constitute a barrier to empowerment and resilience.

### 4.3 Building Resilience

MMR helped to build resilience by *enhancing skillsets* to manage pain in everyday life. The multidisciplinary team supported both physical aspects and psychological strategies. Individuals also learned to prioritise self‐care, maintain a healthy balance in their routines, and recognise when to take breaks and respect their limits. This result is consistent with previous research that highlights the importance of balancing activities and not being overactive (Hållstam et al. 2015; Battin et al. 2022). The implication of such results for the individual is essential, in that the ability to maintain an independent lifestyle, for example, doing household chores, driving a car, and attending social activities, can be deeply adversely affected in those who suffer from chronic pain (Dureja et al. 2013; Harker et al. 2012; Nilsing Strid and Ekelius‐Hamping 2020).

MMR encouraged *proactivity* in life, to build resilience in the face of pain. With increased self‐confidence and openness to new approaches, individual responsibility was recognised as essential for continuing to engage in physical activities and for regulating mindset. These results align with other individuals' experiences of MMR in primary care (Semedo et al. 2020). Engaging in valuable activities is essential for maintaining one's self‐image (Lennox Thompson et al. 2020). As illustrated by a study of women with chronic widespread pain, to have a satisfying life, it is essential not to let pain overshadow one's everyday life (Westergården et al. 2021). The proactivity experienced following MMR may, however, be short‐lived, and future studies should explore its sustainability over time.

Not all participants in the current study developed new skills or became proactive. These divergent experiences are important because they show that MMR in primary care was not experienced as uniformly beneficial. For participants with prior rehabilitation experience, parts of the programme could be perceived as repetitive or too basic, which may have reduced its relevance. Similarly, group discussions characterised by negativity or rumination could hinder rather than support empowerment. These findings suggest that the perceived value of MMR may depend on the match between programme content, group composition, participants' previous experiences, and their expectations of rehabilitation. This raises questions about whether the programme content, group composition, and level of individual tailoring sufficiently matched participants' needs and previous experiences. These findings can be understood in relation to previous research that has problematised patient selection for MMR in primary care and the need to consider which patients are likely to benefit from this form of rehabilitation (Pietilä‐Holmner et al. 2020; Stenberg et al. 2016). Previous negative experiences with healthcare could impact the outcome (Battin et al. 2022), as could limited health literacy and a lack of responsiveness to health literacy (Cesar et al. 2022; Mackey et al. 2016).

### 4.4 Methodological Considerations

In qualitative research, trustworthiness is considered in relation to four established criteria: credibility, dependability, confirmability, and transferability (Bengtsson 2016; Graneheim and Lundman 2004). Credibility was enhanced by making participants' voices audible through a variety of quotations, and to reduce recall effects, the interviews were conducted shortly after the MMR. Since the findings illustrate both positive and negative aspects of MMR delivery, an overrepresentation of more positive experiences is considered small. Despite the small participant number and relatively short interviews, which may have limited the breadth and depth of experiences captured, sample adequacy was considered in relation to information power (Malterud et al. 2016). The focused aim, the specificity of the sample, and the manifest qualitative content analysis supported the material's relevance to the aim. However, a larger, more varied sample could have provided further insights into how prior rehabilitation experiences, pain conditions, expectations, and group composition may shape experiences of MMR.

Even though the data were gathered in 2017–2018, the results remain relevant, as they offer valuable insights into MMR in primary care, which is less commonly provided than in specialist settings in Sweden. In addition, access to MMR in primary care in Sweden has drastically decreased in recent years, while the number of patients with chronic pain who would benefit from it remains. Thus, there is a continued need to explore and report MMR findings in primary care to alert stakeholders and benefit patients.

Dependability was strengthened by using the same opening question at the start of each interview and by probing to encourage elaboration (Graneheim and Lundman 2004). The main author initially conducted the analysis and then discussed with co‐authors throughout the process to ensure consistency (Bengtsson 2016; Graneheim et al. 2017). The interviewer's clinical role in other MMR programmes could constitute bias, but to strengthen confirmability, several researchers were involved in the analysis of the transcribed material, which helped reduce the influence of individual researcher perspectives and ensured that the findings were grounded in data (Brinkmann and Kvale 2015; Graneheim et al. 2017). The single‐site setting limited the number of potential participants who had conducted MMR in primary care, potentially influencing the range of experiences represented. Although limited participant information protects confidentiality, it may also affect transferability. As primary care structures vary across countries, findings may not translate directly to other settings. However, the range of experiences captured supports the relevance of findings to similar primary care settings and to other primary care clinics and stakeholders seeking to improve MMR accessibility.

## 5 Conclusion

MMR provided a framework for addressing chronic pain beyond managing symptoms. It facilitated better communication between patients with chronic pain and HPs, which may contribute to improving health outcomes and individual autonomy.

Importantly, MMR supported a shift from seeing pain as purely physical to grasping and embracing the pain from a more biopsychosocial perspective, while building resilience for a better everyday life. While experienced as effective for many patients with chronic pain, adapting the MMR programme to individual needs and prior experiences could further enhance its efficacy for patients who today drop out or have less benefit.

## Author Contributions

All authors participated in the study's concept and design. Data collection was conducted by J.W., and C.S. led the data analysis and was responsible for writing the manuscript. The analysis and results were compared and discussed with all authors (C.S., J.W., I.L. and S.B.). All authors contributed significantly to the manuscript and participated in its critical review. All authors have read and agreed to the final version of the manuscript.

## Funding

The authors have nothing to report.

## Conflicts of Interest

The authors declare no conflicts of interest.

## Data Availability

The data that support the findings of this study are available on request from the corresponding author. The data are not publicly available due to privacy or ethical restrictions.
